# Persistence of Hepatitis C Virus during and after Otherwise Clinically Successful Treatment of Chronic Hepatitis C with Standard Pegylated Interferon α-2b and Ribavirin Therapy

**DOI:** 10.1371/journal.pone.0080078

**Published:** 2013-11-21

**Authors:** Annie Y. Chen, Marija Zeremski, Ranjit Chauhan, Ira M. Jacobson, Andrew H. Talal, Tomasz I. Michalak

**Affiliations:** 1 Molecular Virology and Hepatology Research Group, Faculty of Medicine, Health Sciences Centre, Memorial University, St. John's, Newfoundland and Labrador, Canada; 2 Center for the Study of Hepatitis C and Division of Gastroenterology and Hepatology, Weill Cornell Medical College, New York City, New York State, United States of America; 3 Division of Gastroenterology, Hepatology and Nutrition, Department of Medicine, State University of New York, Buffalo, New York State, United States of America; University of Washington, United States of America

## Abstract

Resolution of chronic hepatitis C is considered when serum HCV RNA becomes repeatedly undetectable and liver enzymes normalize. However, long-term persistence of HCV following therapy with pegylated interferon-α/ribavirin (PegIFN/R) was reported when more sensitive assays and testing of serial plasma, lymphoid cells (PBMC) and/or liver biopsies was applied. Our aim was to reassess plasma and PBMCs collected during and after standard PegIFN/R therapy from individuals who became HCV RNA nonreactive by clinical testing. Of particular interest was to determine if HCV genome and its replication remain detectable during ongoing treatment with PegIFN/R when evaluated by more sensitive detection approaches. Plasma acquired before (n = 11), during (n = 25) and up to 12–88 weeks post-treatment (n = 20) from 9 patients and PBMC (n = 23) from 3 of them were reanalyzed for HCV RNA with sensitivity <2 IU/mL. Clone sequencing of the HCV 5′-untranslated region from plasma and PBMCs was done in 2 patients. HCV RNA was detected in 17/25 (68%) plasma and 8/10 (80%) PBMC samples collected from 8 of 9 patients during therapy, although only 5.4% plasma samples were positive by clinical assays. Among post-treatment HCV RNA-negative plasma samples, 9 of 20 (45.3%) were HCV reactive for up to 59 weeks post-treatment. Molecularly evident replication was found in 6/12 (50%) among PBMC reactive for virus RNA positive strand collected during or after treatment. Pre-treatment point mutations persisted in plasma and/or PBMC throughout therapy and follow-up. Therefore, HCV is not completely cleared during ongoing administration of PegIFN/R otherwise capable of ceasing progression of CHC and virus commonly persists at levels not detectable by the current clinical testing. The findings suggest the need for continued evaluation even after patients achieve undetectable HCV RNA post-treatment.

## Introduction

Hepatitis C virus (HCV) is a single-stranded RNA virus that is the cause of clinically diagnosable chronic infection in approximately 170 million people worldwide. Of those acutely afflicted, 15% spontaneously resolve hepatitis, while the remaining develop chronic hepatitis C (CHC) [1]. Up to15% of the patients with CHC progress to fibrosis and cirrhosis, and they are at a greater risk of developing hepatocellular carcinoma (HCC) [2]. HCV is infectious even in trace amounts, with approximately 10 virions or 20 copies of viral RNA capable of transmitting infection in chimpanzees [3], [4] and with 20 to 50 virions able to establish productive infection in human T cells *in vitro* [5]. The introduction of nucleic acid amplification assays detecting HCV genomes with high sensitivity, *i.e.*, <10 virus genome equivalents (vge) or copies/ml or <2.5 vge/µg RNA (<2 IU/ml), revealed that HCV persists at low levels (usually below 100 vge/ml) for years after clinical resolution of hepatitis either spontaneously or due to treatment with interferon-α (IFN) alone or pegylated IFN/ribavirin (PegIFN/R) [6], [7]. The long-term consequences of this essentially asymptomatic infection, termed as occult HCV infection (OCI), remains uncertain; however, OCI coincides with histologically evident protracted low grade liver inflammation and fibrosis in some patients for at least 10 years after completion of antiviral treatment [8]–[11]. Also, clinically diagnosed sustained virological response (SVR) achieved due to IFN or PegIFN/R does not universally prevent progression to HCC, which develops in up to 3.9% of these individuals [12]–[17]. Contrary to prevailing opinion based on the currently available clinical testing for HCV RNA, clinical diagnosis of SVR does not reflect molecular eradication of HCV, as evidenced by assays of enhanced sensitivity supplemented with examining of serial samples of plasma, peripheral blood mononuclear cells (PBMC) and, when available, liver biopsies, and by procedures enriching HCV in test material by amplifying viral RNA recovered from larger amounts of serum, liver biopsy material and/or from mitogen-stimulated PBMC [6]–[9], [18]–[20]. Further, the detection of HCV RNA replicative (negative) strand is not uncommon in OCI, particularly when *ex vivo* stimulated PBMC and liver biopsy material are analysed [6], [8], [11], [20]. Since discovery of OCI in 2004, persistence of HCV after SVR was the subject of studies by different groups which delineated virological and some unique immunological properties of this infection [6]–[9], [21]–[23]. Among others, OCI displays a distinct profile of antiviral cytokine expression in PBMC when compared to either CHC or healthy individuals, shows an antagonistic relation between HCV and IFN-α expression in PBMC, and that HCV replication in this compartment can be completely eliminated by activation of endogenous IFN-α [22], [23]. Nonetheless, OCI is rarely investigated and knowledge on this subject remains incomplete. To broaden characterization of this infection entity, in particular to learn about the fate of HCV during and shortly after completion of otherwise clinically successful treatment with PegIFN/R, we re-examined, using highly sensitive HCV genome detection methods, serial plasma and, in some cases, PBMC samples collected prior to, during and after completion of PegIFN/R therapy from patients with CHC who finally achieved clinical SVR.

## Materials and Methods

### Ethics Statement

The study was approved by the Weill Cornell Medical College institutional review board and was performed in accordance with the Declaration of Helsinki. The samples were collected after signing written informed consent.

### Patients and samples

Serial plasma samples (n = 56) from 9 patients (3 men and 6 women; ages 38 to 62), who clinically resolved CHC in response to treatment with PegIFN/R, and sequential PBMC samples (n = 23) from 3 of them were investigated (Table 1). The patients were infected with HCV genotype 1 or 2 (Table 1). The origin and the route of HCV infection were undetermined; however none of the patients was an active drug user during treatment or follow-up. None of them also was co-infected with hepatitis B virus (HBV) or human immunodeficiency virus or was receiving immunosuppressive or anti-cancerous therapy. All patients received PegIFN/R treatment for 24 or 48 weeks (wks) with the exception of 6/F, 7/F and 2/F who were treated for 25, 44 or 68 wks, respectively (median treatment time for all 9 patients was 43.3 wks) (Table 1). The therapy resulted in the decline of plasma HCV RNA to undetectable levels, as measured by clinical laboratory tests (see below), and in normalization of liver enzymes, *i.e.*, alanine aminotransferase (ALT) and aspartate aminotransferase (AST), starting within 3 to 4 wks after initiation of PegIFN/R. In regard to plasma samples, 11 samples from a total of 19 collected prior to initiation of the therapy (pre-treatment samples) were available for re-examination. These 11 samples were obtained between week 21 and one before the start of treatment (median time of collection 8.1 wks). Among 55 plasma samples collected during the treatment period (on-treatment samples), 25 were available for re-evaluation (Table 1). Also, from 34 plasma samples collected during follow-up lasting for up to 88 wks after completion of PegIFN/R therapy (post-treatment samples), 20 were available for reanalysis. The time of the last sample collection from individual patients ranged between 12 and 88 wks post-treatment (median 33.1 wks). Plasma was stored in 1-mL aliquots at −80°C until re-tested. One 1-mL aliquot per sample was available for investigation.

**Table 1:** Clinical characteristics and samples collected from patients investigated.

Patient/sex	HCV genotype	Duration of treatment (weeks)	Follow-up after treatment (weeks)	Plasma samples with treatment (total vs tested)								PBMC samples/phase treatment			
				Before		During		After		Overall		Before	During	After	Overall Tested
				†Total	‡Re-tested	†Total	‡Re-tested	†Total	‡Re-tested	†Total	‡Re-tested				
1/F	1b	36	23	2	1	5	3	4	4	11	8	NA	NA	NA	0
2/F	1b	68	59	2	1	9	1	6	3	17	5	NA	NA	NA	0
3/M	1a	48	12	2	0	9	5	4	1	15	6	NA	NA	NA	0
4/F	1b	48	26	2	1	4	3	2	2	8	6	NA	NA	NA	0
5/M	2b	24	24	1	1	5	2	4	3	10	6	1	2	3	6
6/F	1a	25	18	2	1	4	1	4	1	10	3	NA	NA	NA	0
7/F	2b	44	88	2	1	9	4	5	2	16	7	1	4	2	7
8/F	2a	48	24	4	3	6	4	3	3	13	10	3	4	3	10
9/M	2b	48	24	2	2	4	2	2	1	8	5	NA	NA	NA	0
Total				19	11	55	25	34	20	108	56	5	10	8	23

F, female; M, Male; NA, not available;

† , Total number of samples collected;

‡ ,Samples available for re-examination.

Serial PBMC samples collected at the time of plasma acquisition were available from 3 of the patients (Table 1). In total, 23 PBMC samples were investigated of which 5 were obtained before, 10 during and 8 up to 88 wks after completion of PegIFN/R treatment. PBMCs were stored in liquid nitrogen at 5×10^6^ to 1×10^7^ cells per vial. One vial per sample was available for this study.

### PBMC isolation and mitogen stimulation

PBMCs were isolated from whole blood by density gradient centrifugation. After washing, cells were suspended in heat-inactivated fetal calf serum (FCS) with 10% DMSO, progressively cooled down to −80°C, and stored in liquid nitrogen. A two-step approach was used to prepare PBMCs for evaluation of HCV expression. In the first approach when approximately 1×10^7^ cells were available for investigation, the cells were thawed, extensively washed, and split to two equal portions. One of the portions was spun down and the cell pellet was immediately subjected to RNA extraction. These cells were designated as untreated. The remaining portion of the cells, as well as PBMC samples that contained approximately 5×10^6^ cells, were suspended in 5 ml of RPMI 1640 medium with 10% FCS, 2 mM glutamine, 50 U of penicillin/ml, 50 µg of streptomycin mL-1, 0.1 mM nonessential amino acids (all from Invitrogen Life Technologies, Burlington, Ontario, Canada), 5 µg/ml of phytohemagglutinin (PHA; Sigma-Aldrich Mississauga, Ontario, Canada) and 20 U/ml human recombinant interleukin-2 (IL-2; Roche Molecular Diagnostics, Pleasanton, CA), and cultured for 72 hours as reported [5], [6], [11]. These *ex vivo* stimulated cells were designated as treated cells.

### RNA extraction and cDNA transcription

Total RNA was extracted from 250 µl of plasma and, if the sample was HCV RNA nonreactive, from the remaining 750 µl of the same sample using Trizol LS reagent (Invitrogen) [6], [11]. Untreated PBMC and those cultured in the presence of PHA and IL-2 (treated cells) were extracted with Trizol (Invitrogen). With each RNA extraction, a mock sample of sterile bi-distilled water (contamination control), serum or PBMC from a healthy donor (negative control) and serum or PBMC from a patient with serum HCV RNA-reactive CHC (positive control) were included. RNA from cells (2 to 4 µg) and all RNA extracted from 250 µl or 750 µl of plasma were transcribed to cDNA with Moloney murine leukemia virus reverse transcriptase (Invitrogen), as reported [6], [11].

### Detection of HCV RNA positive and negative strands

For detection of HCV RNA positive (vegetative) strand, reverse transcriptase-polymerase chain reaction (RT-PCR) was applied using primers specific for the HCV 5′-untranslated region (5′-UTR), cycling conditions and quantification standards reported before [6], [11]. In selected samples (depending upon availability of test material), the presence of the HCV RNA positive strand was also examined by amplification with the virus E2 region-specific primers, as reported [6]. Detection of OCI normally requires two rounds of cDNA amplification by PCR, direct and nested, following stringent precautions typical when working with nested PCR, and inclusion of appropriate contamination and negative controls at each step of RNA and cDNA preparation, and PCR amplification [6], [7]. In all instances, signal specificity was confirmed by nucleic acid hybridization (NAH) using ^32^P-labeled recombinant HCV 5′-UTR-E2 fragment as a probe [6]. Sensitivity of the RT-PCR/NAH assays with either 5′-UTR or E2 region-specific primers was <10 vge/ml or <2.5 vge/µg RNA. Overall, the underlying methodology and the resulting sensitivity of these assays employed in the current study were closely comparable to those of the PCR/NAH assays previously developed and applied for detection of occult HBV and woodchuck hepatitis virus infections [24], [25]. HCV RNA negative (replicative) strand was detected by RT-PCR/NAH using r*Tth* DNA polymerase, as described in detail before [6], [26]. This assay detects ∼10^2^ copies of the correct (negative) strand, while identifying ≥10^6^ vge of the positive strand [6], [26]. Specificity of PCR amplicons and validity of the controls was routinely confirmed by NAH. In every analysis, a number of negative and contamination controls were included, as described before [6].

### Cloning, sequencing and analysis of the HCV 5′-UTR

To assess possible sequence variations and compartmentalization of HCV, 5′-UTR amplicons were cloned using the TOPO-TA cloning kit (Invitrogen). The highly conserved 5′-UTR was chosen because it would allow for reliable identification of most unwavering sequence variants. Ten or 20 randomly selected clones were analyzed from each PCR product derived from 7 pairs of plasma and PBMC samples collected at the same time points of follow-up (with one exception) prior to, during or after completion of PegIFN/R treatment from 2 patients (5/M and 8/F) from whom sufficient amounts of sample material were available. The sequence was determined in both directions using universal forward and reverse M13 primers, and the ABI-Prism 7000 Sequence Detection System (Applied Biosystems, Streetsville, Canada). The resulting sequences were aligned using Sequencher software version 4.7 (Gene Codes Corp., Ann Arbor, MI). The HCV subgenotype-specific sequences D00944 and AF169005 for 2a genotype, and D10988 and AF238486 for 2b genotype from GenBank were used as the references. The phylogenetic relationships of the variant 5′-UTR sequences identified in plasma and PBMC of 5/M and 8/F patients were examined by the maximum-likelihood method [27].

### Statistical analysis

Results were analyzed by Chi-Square test using SPSS Statistics software version 19.0 (IBM, Armonk, New York State, USA). Differences between sample groups were considered to be significant when *P* values were below or equal to 0.05.

## Results

### HCV RNA detection in clinical assay virus-negative plasma samples collected during or after antiviral treatment

Using the standard clinical assays, plasma HCV loads in samples collected prior to initiation of PegIFN/R therapy ranged between 1.5×10^4^ and 2.4×10^6^ IU/ml (mean 4.5×10^5^ IU/ml). All 11 samples available for re-examination were, as expected, HCV RNA reactive when RNA extracted from 250-µl plasma was assayed by RT-PCR/NAH (Table 2). However, among 55 samples collected in total during the treatment period only 3 (5.4%) obtained from 3 different patients were reactive for HCV RNA by the clinical assays at levels not exceeding 1.1×10^3^ IU/ml. When 25 of the 55 samples were re-tested by RT-PCR/NAH using RNA from either 250 µl or 750 µl of plasma, 17 (68%) were identified to be HCV RNA positive (Table 2). These positive samples were obtained at different time points of PegIFN/R treatment from 8 of 9 patients examined, including samples collected at the end or within one month prior to completion of the therapy from 4 of the patients. It should be noted that from 2/F patient who was found HCV RNA nonreactive during therapy only a single plasma sample collected through that period was available for re-examination; however, subsequent plasma samples from the same individual were virus reactive for up to 59 wks post-treatment. Further, amongst 34 post-treatment samples none was HCV RNA reactive by the standard clinical assays. Nonetheless, among 20 of these 34 samples re-examined, which were collected between 18 and 88 wks after treatment, 9 (45%) were HCV RNA positive by RT-PCR/NAH. Thus, virus was detected in 8 of the 9 samples when RNA extracted from 750 µl of plasma was tested (Table 2). These 9 reactive samples originated from 4 of the 9 individuals studied. In these 4 patients, virus reactive samples were collected at 23–24 or 59 wks post-treatment. Overall, using as template RNA extracted from 750 µl of plasma acquired either during or after completion of PegIFN/R therapy gave more than a 3-fold greater rate of HCV RNA detection than in RNA isolated from 250-µl samples when tested by RT-PCR/NAH, *i.e.*, 19/38 (50%) vs. 7/45 (15.5%), respectively (*P* = 0.001) (Table 2). Overall, among 45 on- or post-treatment samples re-examined, of which only 3 (6.6%) were HCV RNA reactive by clinical assays, 26 (57.7%) were positive for virus by more sensitive RT-PCR/NAH, giving in total an 8.7-fold increase in detection of plasma HCV RNA (*P*<0.0001).

**Table 2:** HCV RNA detection in plasma samples found negative by clinical laboratory assays and in PBMC of patients who resolved CHC due to PegIFN/R treatment.

	Plasma HCV RNA positivity (%)				PBMC HCV RNA positivity (%)			
Phase of treatment	Samples tested	250 µl	750 µl	Total HCV RNA positivity (%)	Samples tested	Untreated (naive)	Treated (stimulated)	Total HCV RNA positivity (%)
Before	11	11	NA	11/11 (100%)	5	1	4	5/5 (100%)
During	25	6	11	17/25 (68%)	10	4	4	8/10 (80%)
After	20	1	8	9/20 (45%)	8	1	3	4/8 (50%)
Total	56	18	19	37/56 (66%)	23	6	11	17/23 (73.9%)

NT, not tested.

### Expression of HCV RNA in PBMC of CHC patients prior to, during and after antiviral treatment

In the first instance, when a cell number of about 1×10^7^ was available for investigation, PBMC (*i.e.*, untreated cells) were directly subjected to RNA extraction and HCV RNA expression was examined. As showed in Table 2, 6 of 23 (26.4%) samples tested under such conditions were HCV RNA positive strand reactive. Taking advantage of the previous finding that mitogen stimulation of lymphoid cells from HCV-infected patients enhanced virus replication and, in consequence, virus detection [6], [11], [18], [20], the initially HCV RNA-negative PBMC samples or those in which the cell number was close to 5×10^6^ were cultured in the presence of PHA and IL-2 and then HCV RNA expression assessed. This *ex vivo* stimulation allowed detection of HCV in an additional 11 (*i.e.*, 11/17; 54.7%) samples. Overall, virus was identified in 17 of 23 (73.9%) PBMC samples tested (Table 2). Among them, 8 of 10 (80%) obtained during PegIFN/R treatment and 4 of 8 (50%) acquired after completion of therapy were HCV RNA positive.

### Evidence of HCV replication in PBMC of patients on and after anti-HCV therapy

**Figure 1:**
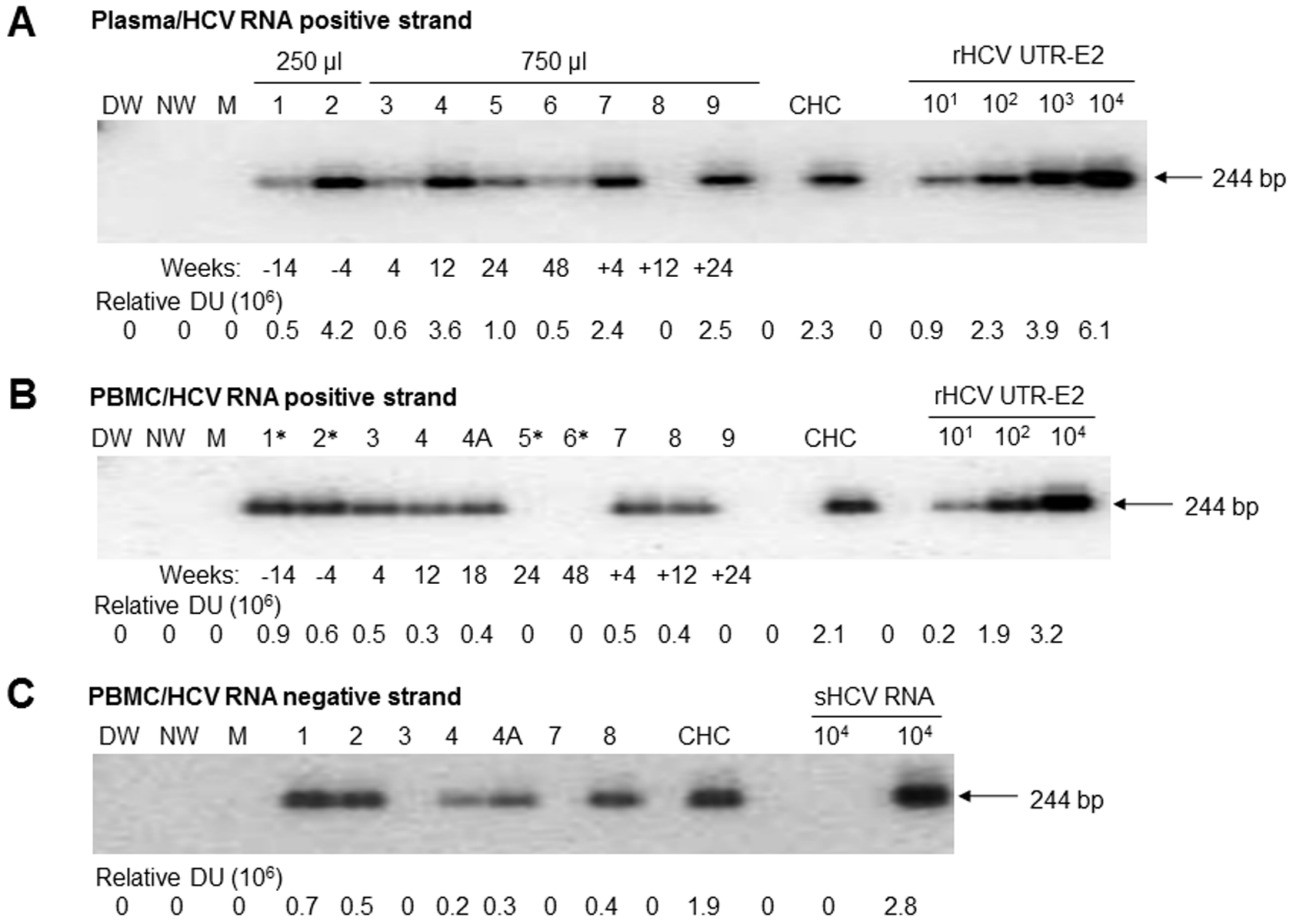
Expression of HCV RNA positive strand in serial plasma and PBMC samples and HCV RNA negative (replicative) strand in PBMC prior to, during and after completion of PegIFN/R treatment of 8/F patient with the initial diagnosis of CHC. (A) HCV RNA positive strand detection using total RNA extracted from 250 µl or 750 µl of plasma. (B) HCV RNA positive strand identification using 2 µg of total RNA extracted from either mitogen-treated (*) or native (untreated) PBMC. (C) Detection of HCV RNA negative strand in HCV RNA positive strand reactive PBMC samples shown in B. Plasma and PBMC were collected in parallel (except 4A PBMC sample) at the time points (weeks) indicated under panels A and B. Minuses before week numbers indicate sample collections prior to initiation of PegIFN/R therapy, while pluses indicate collections after completion of the treatment. As positive controls for HCV RNA positive strand detection, RNA extracted from equivalent of 10 µl of HCV RNA-positive plasma (panel A) or 1 µg RNA from PBMC (panel B) of a patient with active CHC, and serial 10-fold dilutions of recombinant HCV 5′-UTR-E2 (rHCV UTR-E2) fragment carrying indicated copy numbers/reaction were used. For HCV RNA negative strand detection, 2 µg of total RNA from PBMC of the same control CHC patient as in panels A and B, and synthetic HCV RNA positive strand (sHCV RNA pos) and HCV synthetic RNA negative strand (sHCV RNA neg) at 10^4^ copies/reaction were used as positive and specificity controls. Water amplified in direct (DW) and nested (NW) reactions and a mock (M) extraction served as contamination controls. Positive signals showed the expected 244-bp oligonucleotide fragments. Numbers under the panels represent relative densitometric units (DU) given by hybridization signals.

All PBMC identified to be HCV RNA positive strand reactive were evaluated for HCV RNA negative (replicative) strand using the highly sensitive RT-PCR/NAH assay [6], [11], [26]. Since this replication intermediate normally occurs at a much lower copy number than the positive strand and the assay detecting the negative strand is approximately 10 to 100-fold less sensitive than that used for the positive strand identification [6], [26], HCV RNA negative strand expression was examined only in PBMC found reactive for the positive strand. Overall, this form of HCV RNA was detected in 10 out of 16 (62.5%) PBMC samples investigated (Table 3 and Fig. 1C). Among the negative strand reactive PBMCs, 4 out of 4 samples were collected prior to treatment, 4 of 8 during ongoing therapy, and 2 of 4 post-treatment (Table 3). The reactive post-treatment samples were obtained at 12 wks after cessation of PegIFN/R therapy.

**Table 3:** Detection of HCV RNA positive strand in plasma and HCV RNA positive and negative (replicative) strands in parallel PBMC samples from patients who clinically resolved CHC due to PegIFN/R treatment.

			HCV RNA positive strand			HCV RNA negative strand
			Plasma	PBMC		
Patient	Week of treatment	Phase of treatment	250 µl or 750 µl	Untreated (naive)	Treated (stimulated)	PBMC
5/M	−5	Before	POS	NT	POS	POS
	4	During	POS	POS	NT	POS
	24	During	NEG	POS	NT	NEG
	+4	After	POS	NT	POS	NEG
	+12	After	POS	NT	POS	POS
	+24	After	POS	NEG	NEG	NA
7/F	−14	Before	POS	NT	POS	POS
	4	During	POS	NEG	POS	NEG
	25	During	NEG	NEG	POS	POS
	29	During	NEG	NEG	POS	NEG
	44	During	POS	NT	NEG	NA
	+6	After	NEG	NEG	NEG	NA
	+88	After	NEG	NEG	NEG	NA
8/F	−14	Before	POS	NEG	POS	POS
	−4	Before	POS	NT	POS	POS
	4	During	POS	POS	NT	NEG
	12	During	POS	POS	NT	POS
	18	During	No sample	POS	NT	POS
	24	During	POS	NEG	NEG	NA
	48	During	POS	NT	NEG	NA
	+4	After	POS	POS	NT	NEG
	+12	After	NEG	NT	POS	POS
	+24	After	POS	NT	NEG	NA

M, male; F, female; −, prior to initiation therapy; +, after completion of therapy; POS, positive; NEG, negative; NT, not tested; NA, not available.

### Detection of HCV in paired plasma and PBMC samples during and after antiviral treatment

Examination of HCV expression in serial plasma and PBMC samples collected at the same time points of follow-up was possible in 3 of the patients investigated. Considering HCV RNA detection in either 250-µl or 750-µl plasma samples and in either untreated or *ex vivo* stimulated lymphoid cells (Table 3), among 22 plasma-PBMC pairs examined, 13 (59.1%) were HCV positive in both plasma and PBMC, while virus was detected in 5 (22.7%) of the pairs in plasma only and in 4 (18.2%) other pairs in PBMC alone. Further, among 18 plasma-PBMC pairs collected during the treatment or post-treatment periods, 9 (50%) plasma-PBMC pairs were HCV positive in both compartments, while 5 (27.8%) and 2 (22.2%) were HCV reactive in plasma and PBMC, respectively.

Identification of HCV RNA in serial plasma and PBMC samples collected at the same time points of follow-up from 8/F patient infected with HCV genotype 2a and treated for 48 wks with PegIFN/R was shown in Figures 1A and 1B, respectively. Figure 1C illustrates detection of HCV RNA replicative strand in PBMC samples identified to be virus RNA positive strand reactive.

### Persistence of HCV variants in plasma and PBMC during and after antiviral therapy

To assess whether otherwise successful treatment of CHC had an effect on the diversity of HCV variants, clones of 5′-UTR amplicons derived from 5/M and 8/F plasma and PBMC collected before, during and after completion of PegIFN/R administration were sequenced in both directions. Two types of comparisons were performed; one using as the baseline the HCV sequence identified in plasma of a given individual prior to initiation of therapy and second using as references two relevant sequences from GenBank for each of the HCV subgenotype of interest. The analyses showed that the point mutations unique to a given individual already existed in the pre-treatment plasma and/or PBMC, that the majority of these mutations persisted through therapy and follow-up, and that some variants tended to spread with time from one compartment to another, *i.e.*, from plasma to PBMC (*e.g.*,126insC, G151A, A256G and C271T in 58/F) or vice versa (*e.g.*, G97A and A115G in 58/F) (Table 4). Considering the HCV sequence identified in the pre-treatment plasma, the results indicated that HCV variants unique to PBMC appeared prior to as well as during treatment and some of them persisted through the whole observation period. It is of note that two point mutations, G255T and A275C, which were consistently detected in all clones tested (n = 100) from plasma or PBMC of 8/F, were distinct from the 2b genotype sequences reported in GenBank used for comparison (data not shown). Overall, the analysis confirmed that HCV not only persisted but also suggested that its replication progressed during the period of administration of PegIFN/R since new variants appeared and spread from one compartment to another. The 5′-UTR variants and wild-type 5′-UTR sequences found in 5/M and 8/F patients have been deposited in GenBank under accession numbers KF548005-KF548035.

**Table 4:** Single-nucleotide polymorphisms in the HCV 5′-UTR sequence in sequential plasma and PBMC samples obtained from patients with CHC prior to, during and after treatment with PegIFN/R.

			Phase of treatment				
Age/sex	Genotype	Sample	Before	During	After		
Time of collection (week):			−5†	4†	+12†		
5/M	2b	plasma	A177G (1)	A177G (1)	A177G (2)		
		PBMC	A177G (1)		A177G (1)		
Time of collection (week):			−4‡	12†	+4†	+12†	+24†
8/F	2a	plasma	*126insC (1)*	**A115G** (1)	**A115G** (1)	NA	**A115G** (1)
			G151A (2)	*126insC (1)*	*126ins (2)*		*126insC (1)*
			A256G (1)	C236T (1)	**C271T** (2)		**C271T** (1)
			**C271T** (1)	**C271T** (1)			
		PBMC	G97A (1)	*126insC (1)*	*126insC (2)*	*126insC (2)*	NA
			**A115G** (3)	125delC (1)	**C271T** (2)	**C271T** (2)	
				A256G (1)			
				**C271T** (1)			

M, male; F, female; PBMC, peripheral blood mononuclear cells; −, prior to initiation of therapy; +, after completion of therapy; NA, not available;

† , 10 clones per sample sequenced;

‡ , 20 clones per sample sequenced.

**Figure 2:**
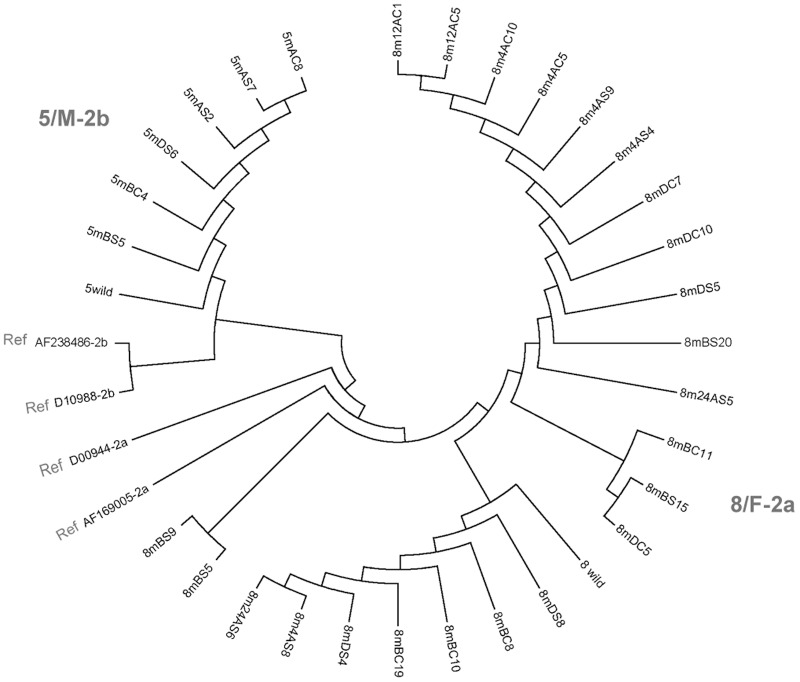
Phylogenetic analysis of the HCV 5′-UTR variants found in plasma and PBMC from patients 5/M and 8/F obtained prior to, during and after PegINF/R therapy. The numbers 5 and 8 identify patient 5/M and 8/F, respectively. M indicates the variant and wild wild-type sequence found in the majority of the clones derived from a given patient prior to, during and after therapy. B stands for before treatment, D during treatment, and A after treatment with PegIFN/R. The variants from plasma are marked with S, while those from PBMC with C. The numbers 1–20 indicate individual clones. The genotype 2a and 2b nucleotide sequences serving as references are marked as Ref.

The phylogenetic tree analysis of the variant 5′-UTR sequences identified in 5/M and 8/F showed the lack of separate clustering of plasma and PBMC derived variants (Fig. 2). This was consistent with the finding that the same variants occurred both in plasma and PBMC, and that they tended to spread from one compartment to another during follow-up (Table 4).

## Discussion

This study demonstrated that HCV is not cleared during ongoing administration of standard PegIFN/R therapy or after its completion even when the treatment lowers circulating HCV to the levels undetectable by clinical testing and was finally capable of inhibiting progression of CHC. While persistence at low levels of HCV in plasma or sera in the context of detectable antibodies to HCV (anti-HCV), undetectable serum HCV RNA by clinical testing, and essentially normal levels of liver enzymes has been reported in individuals long after clinical resolution of CHC following treatment with IFN or PegIFN/R [5], [6], [8], [9], [11], [20], the detection of HCV and its replication during ongoing treatment with PegIFN/R when the virus genome became undetectable by clinical assays has not yet been examined and our study is the first in this regard. Although this finding *per se* was expected, considering frequent detection of OCI after clinically apparent SVR when methods enhancing virus identification were used [6], [11], [18], [19], [20], it adds a new dimension to the robustness of HCV infection in the face of antiviral treatment even if such treatment is ultimately capable of inhibiting progression of the disease. It also confirms inability of the standard PegIFN/R therapy to completely eradicate HCV from an infected host.

In addition to persistence of HCV in plasma during OCI, virus and its replicating genomes were uncovered in PBMC and liver biopsies in individuals for many years after having been considered to be clinically cured of hepatitis C [5]–[9], [11], [20], [22]. In regard to infection of PBMC, different subsets of circulating immune cells were found to carry HCV, its RNA replication strand, displayed intracellularly virus protein (*i.e.*, nonstructural protein 5a; NS5a), and virus sequence variants not encountered in the patients' plasma or liver [8], [20], [28]–[30]. Also, cultured PBMC from individuals with OCI continuing after SVR due to PegIFN/R therapy released HCV virion-like particles identifiable by immunoelectron microscopy with anti-HCV E2 antibodies and transmitted infection to virus-naïve human T lymphocytes in culture [5]. Taken together, the accumulated data showed that HCV residing in immune cells fully retains biological competence, including infectivity [5], [31]. These and other findings prove that immune cells, including T lymphocytes, are targets of naturally occurring virus, they are reservoirs of replicating HCV regardless of symptomatic or occult appearance of infection and, since these cells are readily accessible, they can be utilized for evaluation of the status of HCV replication during infection and progression of antiviral treatment [32]. In this context, the detection of HCV positive and, in some cases, virus negative (replicative) strands in PBMCs in the current study is indicative that these cells constituted a virus reservoir during both ongoing PegIFN/R treatment and after its completion.

In regard to the above, it is of interest to note that human recombinant IFN-α 2b is capable of total elimination of HCV replication in cultured normal human T lymphocytes infected *de novo* with different HCV genotypes at concentration of 1000 U mL-1 [5], [31; MacParland and Michalak, unpublished data]. Also, replication of HCV in T cells of patients with CHC or OCI persisting after SVR can be completely abrogated by activation of endogenous IFN-γ in CHC but of IFN-α in OCI after *ex vivo* stimulation of the cells with a mitogen prompting proliferation of T cells [23]. These data suggest that IFN-α has in fact ability to totally purge HCV from infected cells, at least in the immune cell compartment.

The bidirectional sequencing analysis of the 5′-UTR encompassing the virus internal ribosomal entry site (IRES) suggested nucleotide polymorphisms between plasma and PBMC obtained at the given time points of follow-up, either before, during or following PegIFN/R treatment, which were particularly apparent in samples from 8/F patient (Table 4). The existence of HCV polymorphisms in PBMC comparing with plasma and liver has been previously reported in patients with CHC or OCI and in individuals after SVR with a past exposure to HBV [11], [20], [28], [29]. In this study, a minority of variants identified before or during therapy were not detectable thereafter, suggesting that the breadth of HCV polymorphism tended to decline after cessation of the therapy. However, as already noted, the majority of the point mutations detected prior to the initiation of PegIFN/R therapy persisted throughout the treatment and the post-treatment periods. Moreover, they showed an inclination to spread from one compartment of virus occurrence to another, implying that the antiviral treatment was without meaningful effect on their fate. This trend to spread from PBMC to plasma or vice versa was confirmed by the phylogenetic tree analysis of the 5′-UTR sequences identified during the observation period of 5/M and 8/F patients, which showed the lack of segregation to separate clusters of the variants detected in plasma and PBMC (see Fig. 2). Some of the variants detected were reported before. Thus, 126insC mutation found in plasma of 8/F prior to treatment and then in PBMC and plasma during and after PegIFN/R therapy has been reported for HCV derived from brain [33], while 125delC detected in PBMC during treatment of 8/F patient was previously found in PBMC of a patient with occult HCV infection [11].

Treatment with PegIFN/R inhibits progression of CHC in 40% to 50% of those chronically infected with HCV genotype 1 without benefiting the remaining infected with this genotype [34]. Nonetheless, HCV persists for years, if not decades, even in those who achieved clinical SVR at levels which are only occasionally detectable by clinical molecular tests of improved sensitivities [5], [6], [8], [9], [35]. The relevance of this low level HCV persistence to virological relapse after SVR, as defined by detection of HCV RNA by clinical assays, appears to be low or very low based on the data from pertinent clinical trials and related prospective studies. These data indicate that the late reappearance of HCV detectable by clinical tests among responders to IFN or PegIFN/R is ranging from that close to none to 11.6% [36]–[38]. However, the results outside clinical trials show that virological and clinically evident relapse after IFN-induced SVR happens and is usually linked to situations where the host's immune system is compromised due to either immunosuppressive treatment or comorbid disease [39]–[41]. The accumulated data imply that it will be prudent to monitor the molecular markers of HCV infection and liver function enzymes in patients with a history of HCV infection and SVR who are subjected to temporal or prolonged immunosuppressive therapies and/or suffer from diseases diminishing the host's immune surveillance, as it has become common in recent years for individuals with a past exposure to HBV and persistent occult HBV infection [42], [43].

Currently PegIFN/R treatment remains the most commonly utilized approach against CHC, although patients infected with HCV genotype 1 benefit from supplementing this therapy with direct acting antivirals (DAAs) targeting virus proteases or polymerase. While it is expected that new generations of DAAs will replace the need for IFN-α use in the majority of patients infected with different HCV genotypes, although IFN-α may remain as part of the treatment scheme for patients who relapse after DAA therapy. Nonetheless, to fully recognize the sterilizing potency of new DAAs in the context of the extremely high mutagenic capacity of HCV and the virus' resulting ability to generate drug resistant mutants, the DAA effects on virus replication at extrahepatic sites, particularly in immune cells, should be routinely assessed since these cells also are the site of virus active propagation. There are very limited data in this regard. However, telaprevir, an HCV-specific protease inhibitor recently approved for clinical use (VX-950; Vertex Pharmaceuticals, Cambridge, MA) [44], has been shown to totally inhibit replication of native, patient-derived HCV infecting Molt4 T cells at the cell nontoxic concentration of 4 µM, as our recent study demonstrated [26]. This indicates that this DAA has the ability to enter and inhibit propagation of HCV in cells other than hepatocytes.
